# Food waste accounting along global and European food supply chains: State of the art and outlook

**DOI:** 10.1016/j.wasman.2018.07.032

**Published:** 2018-09

**Authors:** Sara Corrado, Serenella Sala

**Affiliations:** European Commission-Joint Research Centre, Directorate D-Sustainable Resources, Bioeconomy Unit, Via Enrico Fermi 2749, I-21027 Ispra, VA, Italy

**Keywords:** Food loss, Food waste, Estimation, Sustainable Development Goal 12, Circular economy, Waste framework directive

## Abstract

Contributing to environmental pollution and resources depletion, food waste represents a considerable inefficiency of the global food system. Within the United Nations Sustainable Development Goal 12.3, countries committed to halve per-capita food waste generated at retail and consumer levels and to decrease food waste along the food supply chain by 2030. Reliable and detailed information on food waste is of utmost importance for the actors of the food supply chain, organizations and governments willing to implement and monitor effective reduction strategies. The present paper is a review of existing studies on food waste generation at the global and European scales and aims primarily at describing and comparing the approaches adopted, and secondarily at analysing their potential in supporting food waste related European interventions and policies. Ten studies were selected among relevant scientific papers and grey literature and their underlying quantification methodologies were systematically analysed. Methodological elements discussed in the paper include type of waste streams captured by estimations, distinction between edible and inedible food waste along the agro-food supply chain, reported units of measure, overall inefficiencies of the food system, and uncertainty of data. Current estimations of food loss and waste generation range between 194–389 kg per person per year at the global scale, and between 158–298 kg per person per year at the European scale. However, further efforts are needed to improve their level of detail and reliability and to foster their support to food loss and waste-related strategies.

## Highlights

• Current studies are based on different methodological approaches and data sources.
• A robust food waste accounting methodology is needed for food waste reduction.
• Food waste ranges: 194 and 389 kg/p/y globally and 158 and 290 kg/p/y in EU.
• Waste management and food security objectives lead to different accounting needs.
• Distinction in edible and inedible is crucial for food waste prevention and food security.

## 1 Introduction

About one third of the food produced for human consumption is currently wasted at the global scale (FAO, 2011). Food waste (FW) generation, happening throughout the entire food supply chain around the globe, is dominated by different dynamics, ultimately associated by the same unsustainable paradigm. Wasting food contributes to environmental pollution as well as to natural resources degradation and depletion, threatening food security (Foley et al., 2011). Therefore, FW is one of the targets of both environmental and food security policies at different scales. According to the United Nations Sustainable Development Goal (SDG) 12.3, per-capita FW at retail and consumer levels should be halved and FW along the entire food supply chain should be reduced by 2030 (UN, 2015). The European Commission, beyond having committed to the SDG 12.3 reduction target on FW, has included FW among the priority areas of the Circular Economy Action Plan, and is committed to define a common EU methodology for FW accounting and to propose relevant indicators (EC, 2015).

Being aware of the amount of FW generated is the first step to support effective prevention and reduction strategies, and to unveil the potential for FW cascading use from a circular economy perspective. Such information, indeed, allows: (i) defining a baseline to monitor FW reduction over time, (ii) identifying the most important FW streams in terms of mass, (iii) prioritising prevention and reduction interventions, and (iv) highlighting which FW flows may undergo a valorisation process in a circular economy perspective (Caldeira et al., 2017).

In the last years, FW quantification has arisen considerable interest, reflected by the increasing availability of data on FW generation along the food supply chain at various geographical scales. At international level, in 2016, a multi-stakeholder partnership delivered a guidance for quantifying food and associated inedible parts removed from the food supply chain (Hanson et al., 2016). The project FUSIONS (Food Use for Social Innovation by Optimising Waste Prevention Strategies) (FUSIONS, 2016), carried out between 2012 and 2016 and founded by the 7th Framework Program of the European Commission, represents a milestone for FW accounting. Two of the main outcomes of the project were a manual on FW quantification (Tostivint et al., 2016), and an estimate of FW generated at the European level (Stenmarck et al., 2016). Roodhuyzen et al. (2017) made a comprehensive review on definitions and approaches for research on FW, but they excluded quantitative considerations from their study. Xue et al. (2017) made a broad review of existing literature on FW quantification, including an analysis of the bibliometric characteristics, and the assessment of advantages and disadvantages of methods used to measure FW. They found that most of the studies on FW generation were based on literature data and statistics. However, relying on such sources of data may undermine the robustness of resulting considerations. Indeed, the underlying definitions of FW, the system boundaries, and the methods for data collection have a considerable influence on FW quantification (Bräutigam et al., 2014). Furthermore, Gustavsson et al. (2013) highlighted that the FW quantification study by the Food and Agriculture Organization of the United Nations (FAO, 2011), often taken as a reference in subsequent studies (Xue et al., 2017), included several assumptions due to lack of data. Methodological gaps, particularly concerning the accounting of liquid FW and fractions of FW used to feed animals, were emphasised by Møller et al. (2014). Combining these elements, and the fact that data are in some cases outdated (Parfitt et al., 2010), Xue et al. (2017) pointed out the potential scarce representativeness of literature data for specific countries or food commodity groups.

The primary aim of the present paper is to describe and compare the approaches adopted by different methods to account for FW generation as well as their implications on the results. The analysis was performed at the global and European scales because of data availability and the existence of studies based on different methodological approaches. Secondarily, it aims at discussing the potentialities of these methodological approaches in supporting European interventions and policies on FW.

In literature, there are different definitions of the terms food loss and food waste. For the purposes of the present paper, the term FW is intended to include all the food streams, encompassing edible and inedible fractions, leaving the food supply chain, at any stage, from production to consumption.

## 2 Materials and methods

**Fig. 1:**
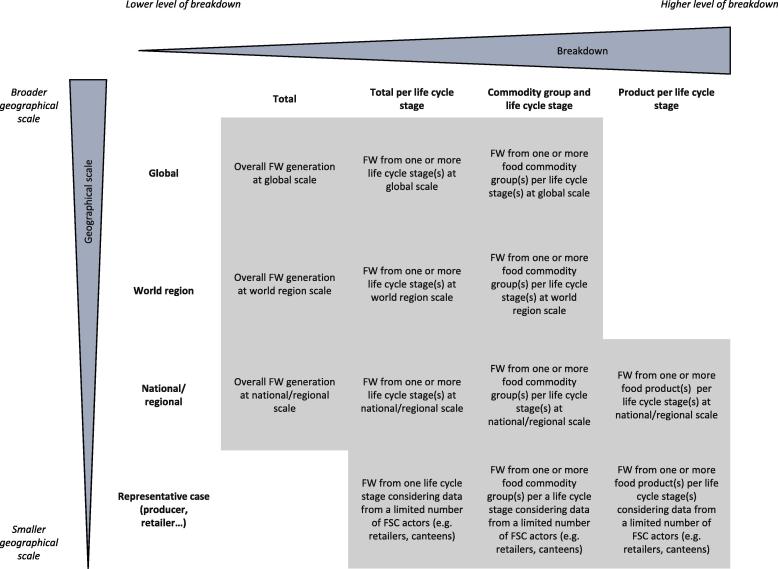
Overview of existing studies on FW accounting. Modified from Corrado and Sala (2018).

According to their scope, FW estimations may report data for different geographical scales and levels of details in term of breakdown of the supply chain (Fig. 1). The focus of the present study is on the global and the European scales. Hence, national studies were excluded from the analysis because the focus was on FW accounting methodologies, considering as well the effects of methodological choices on results. Including national studies would have added variability in the results due to different socio-economic and cultural contexts, limiting the possibility to compare methodologies in light of the results of the accounting.

A literature review has been performed using the bibliometric database Scopus (www.scopus.com). Preliminarily, a screening of the documents including the keywords “food loss” AND “Europe” OR “EU”, “food waste” AND “Europe” OR “EU”, “food loss” AND “global”, “food waste” AND “global” within the title, abstract or keywords was done. The search was bound to papers published or available in Scopus database from January 2005 and June 2017. A refinement of the selection of papers was accomplished, considering titles and, if necessary abstracts, according to the following criteria: (i) the study reported an estimation of FW generated either at European or global scales, based on statistics or proxies; (ii) the study included an overall estimation of FW and it did not focus on a single product; (iii) the estimation interested at least one of the life cycle stages from food manufacturing and consumption; (iv) the amount of FW was expressed in terms of mass. Furthermore, since a large amount of data on FW is reported within scientific reports, we explored as well the grey literature on the topic starting from the analysis of the reference lists reported in selected documents, adopting the abovementioned selection criteria.

The selected studies were reviewed on the basis of elements identified within the FW quantification manual of the FUSIONS project to assess the quality of existing FW estimates (Tostivint et al., 2016), complemented with other relevant aspects. The review focused on: aims of the studies, FW definitions, data sources and quantification approaches, breakdown in product groups, and reliability of estimates. Finally, the results for each stage of the food supply chain were analysed and compared. For such purpose, the results were expressed on a per capita basis considering the global or European population reported respectively in FAOstat (FAO, 2017) and Eurostat (2017a) for the year of estimation. Furthermore, the breakdown of the food supply chain in the following stages was considered: primary production (including post-harvest), manufacturing, distribution, and consumption.

## 3 Results

The keywords research in Scopus database led to the identification of 480 peer-reviewed papers. Among these, five peer-reviewed papers (Table 1) were shortlisted for the analysis according to the selection criteria. From the analysis of the reference lists, five additional scientific reports were included in the review. All of them were published from 2010 onwards, confirming the rapidly growing interest for FW.

**Table 1:** Summary of the reviewed studies and main characterising elements.

Study	Type of document	FW definition or other relevant definitions for the study	Reference year for estimation	Geographical boundaries
Monier et al. (2010)	Report	**Bio-waste**: biodegradable garden and park waste, food and kitchen waste from households, restaurants, caterers and retail premises, and comparable waste from food processing plants. It does not include forestry or agricultural residues, manure, sewage sludge or other biodegradable waste (e.g. naturale textile, paper or processed wood) (EC, 2008) **Food waste**: part of biowaste, composed of raw or cooked food materials. It includes food materials discarded at anytime between farm and fork; in households relating to food waste generated before, during other food preparation, e.g. vegetables peelings, meat trimmings, spoiled or excess ingredients or prepared food	2006	EU27
FAO, 2011, Gustavsson et al., 2013)	Report	**Food losses**: decrease in food quantity or quality in the early stages of the food supply chain, reducing the amount of food suitable for human consumption. Often related to post-harvest activities with lacking system or infrastructural capacities **Food waste**: discarding of food products that are fit for consumption or fit to proceed in the food supply chain. Mostly occurs at the later stages of the food supply chain, such as retail and consumer households	2007	Global (7 world regions)
Bräutigam et al. (2014)	Scientific paper	Not reported, based on FAO definitions	2006	EU27
Vanham et al. (2015)	Scientific paper	Not reported	Average 1996–2005	EU
Porter et al. (2016)	Scientific paper	**Food loss**: referred to the upstream stages (agricultural production, storage and handling, processing) **Food waste**: referred to the downstream stages (distribution, consumer)	From 1961 to 2011 (we considered 2011)	Global (7 world regions)
Stenmarck et al. (2016)	Report	**Food waste**: fractions of food and inedible parts of food removed from the food supply chain to be recovered or disposed (including composted crops, crops ploughed in/not harvested, anaerobic digestion, bioenergy production, co-generation, incineration, disposal to sewer, landifill or discarded to sea)	Mainly 2012	EU
Alexander et al. (2017)	Scientific paper	**Agricultural production losses:** occur during the production process. Include agricultural residues (e.g. roots and straw), unharvested crops and the losses during harvest **Livestock production losses and inefficiencies**: due to the conversion of feed and grass into animal products **Handling, storage and transportation losses**: due to spillage and degradation during storage and distribution. Occur for primary crops, processed commodities and animal products **Processing losses**: occur during the processing of commodities **Consumer waste**: occur between the moments in which the food reaches the consumer and it and is eaten **Over-consumption**: the additional food intake over that required for human nutrition	2011	Global
Eurostat (2017b)	Presentation	Not reported	2012	EU28 (Participation of Member States on voluntary basis).
Tisserant et al. (2017)	Scientific paper	**Solid waste**: any solid output from a human activity that remains inside the technosphere and that requires further treatment before it can be released to the environment or be used as a substitute for other industrial products	2007	Global (48 world regions)
Kemna et al. (2017)	Report	**Food waste:** based on Fusions definition	2011	EU

### 3.1 Aim(s) of the studies

Four of the investigated studies have multiple aims, however we focused on those that drove to the FW quantification exercise for the purpose of the present paper.

The study by Monier et al. (2010) was the first attempt to assess the amount of FW in the European Union. The study by FAO (2011) had the same purpose but at the global scale, including a breakdown in 7 world regions. A critical analysis of the two aforementioned studies was performed by Bräutigam et al. (2014), who compared the results with their own calculations for Europe. Eurostat launched in 2012 the “food waste plug-in” initiative to explore which FW data could be gathered within the data collection framework defined by the Waste Statistics Regulation (Eurostat, 2017b). This paper reports preliminary results for 2012 presented by Eurostat (2017b) at the European Platform on Food Losses and Food Waste (EC, 2017a). Vanham et al. (2015) estimated the amount of water and nitrogen resources lost with European consumer FW. Porter et al. (2016) analysed the variations in FW generation over the years between 1961 and 2011. The report by Stenmarck et al. (2016) was a deliverable of the FUSIONS project on the estimation of FW generation in the European Union. Tisserant et al. (2017) quantified the volume of solid waste generated globally with a breakdown in 46 world regions and 11 waste categories, including FW. The study was performed using a model built on the input-output database Exiobase v2 (Merciai et al., 2014). Alexander et al. (2017) quantified loss, inefficiencies and waste in the global food system, whereas Kemna et al. (2017) made a detailed estimation of food flows in Europe with a focus on the amount of FW generation and the amount of refrigerated food products.

The geographical scale of the assessment was different in the analysed studies (Table 1). Four of them referred to the global level. FAO, 2011, Porter et al., 2016 performed also a breakdown for seven world regions, including Europe and Russia. Tisserant et al. (2017) reported the share of FW generated in 48 world regions, including all the EU-27 Member States. Five studies, instead, focused on the European Union, composed by either 27 or 28 Member States, according to the year in which the estimation was carried out.

### 3.2 FW definitions

Definitions of FW adopted in the analysed studies differed both for the choice of terminology and for the types of materials and destination associated to each term (Table 2).

**Table 2:** Summary of the main characterising elements of FW definitions adopted within the selected studies. X means that the element is considered in the study.

Study	Definition	Type of material				Destination	
	Distinction food loss/waste	Edible fraction	Inedible fraction	Liquid waste	Distinction avoidable/unavoidable	To waste facilities	To feed and bio-refineries
Monier et al. (2010)		X	X			X	
FAO, 2011, Gustavsson et al., 2013)	X	X		X (milk)		X	X
Bräutigam et al. (2014)		X		X (milk)		X	X
Vanham et al. (2015)		X	X	X (milk, alcoholics)	X	X	
Porter et al. (2016)	X	X	X			X	X
Stenmarck et al. (2016)		X	X	X		X	
Alexander et al. (2017)	X	X	X			X	
Eurostat (2017a)		X	X			X	
Tisserant et al. (2017)		X	X			X	
Kemna et al. (2017)		X	X	X	X	X	

The terminology used to refer to FW was explicitly defined in all the studies except Bräutigam et al., 2014, Vanham et al., 2015, Eurostat, 2017b.

FAO (2011) considered the distinction between food losses and food waste. Food losses are intended as a decrease of quantity or quality in the primary stages of the supply chain mostly due to post-harvest activities with lacking system or infrastructural capacities, whereas FW consists in the discard of food that fits for human consumption mainly at distribution and in households. The same distinction was adopted by Alexander et al., 2017, Porter et al., 2016. All the other studies used the term FW referring to the entire supply chain, but with different meanings.

Loss of food at primary production was considered as FW in all the studies, except from the one by Monier et al. (2010) in which data related to primary production were reported but considered out of the scope of the study, and Vanham et al. (2015), who focused their analysis on the consumption stage.

Eight out of ten studies accounted for both edible and inedible parts of food lost or wasted along the supply chain, whereas FAO, 2011, Bräutigam et al., 2014 considered only the edible fractions. Vanham et al., 2015, Kemna et al., 2017 were the only ones accounting separately for avoidable and unavoidable FW at consumption stage, classifying as unavoidable the parts of wasted food considered unfit for human consumption by the intended users, and, vice versa, as avoidable the FW fractions retained apt for human consumption (Kemna et al., 2017). Furthermore, Kemna et al. (2017) reported the amount of FW that was used as animal feed, but they did not consider it as part of FW. Beside discarded food, Alexander et al. (2017) included in their study the amount of agricultural residues and unharvested crops, animal metabolism, and over-eating.

The fraction of liquid FW, such as milk or beverage, is generally disposed via the sewer and not captured by quantification approaches based on waste statistics, such as the one adopted by Monier et al. (2010). The fraction of liquid FW generated along the food supply chain was explicitly considered in the study by Stenmarck et al., 2016, Kemna et al., 2017 and was partly accounted by Vanham et al. (2015) who considered FW of milk and alcoholic beverages, and Bräutigam et al., 2014, FAO, 2011, Porter et al., 2016, who included milk FW.

Moreover, the selection of the materials to be included in the accounting depended as well on their destination (Chaboud and Daviron, 2017). Particularly, two main approaches were identified: (i) including food intended for human consumption, which eventually is not eaten by humans (Bräutigam et al., 2014, FAO, 2011, Porter et al., 2016); (ii) including only food which is sent to waste management facilities, such as incineration, anaerobic digestion and composting (Alexander et al., 2017, Monier et al., 2010, Eurostat, 2017b, Stenmarck et al., 2016, Tisserant et al., 2017, Kemna et al., 2017). The first approach implies the accounting of the FW streams valorised as resources, which is excluded from the second.

### 3.3 Data sources and quantification approach

The quantification of FW can be based on either direct or indirect measurements, derived from secondary data. Direct measurements are generally requiring more resource, and, therefore, applied to single stages of the supply chain, involving a limited number of actors in data collection. On the contrary, indirect measurements best adapt to broader boundaries of analysis (van der Werf and Gilliland, 2017), but may imply higher uncertainties and their accuracy depend on the quality and representativeness of the sources of data (Xue et al., 2017). The ten selected studies were all based on indirect measurements, but different quantification approaches were adopted and different sources of data were considered.

Two studies (Eurostat, 2017b, Monier et al., 2010) were based on data collected by Eurostat, in which data on waste contain a breakdown into 3 digit-waste categories according to the 4-digits European Waste Classification for statistical purposes (EWC-Stat) (EC, 2010). EWC-Stat does not disaggregate the share of FW, which is to different extents included in the waste categories together with other bio-waste streams, such as e.g. garden and park waste. Monier et al. (2010) dealt with this issue refining or substituting Eurostat numbers with national data, when available. Within the food waste plug-in exercise, Eurostat (2017a) collected data on a more detailed level than what is ordinarily done, following the administrative classification List of Waste (LoW), for which a conversion table from the substance oriented classification EWC-Stat exists (EC, 2010). LoW codes were classified according to the fact that they contain, not contain, or partly contain FW.

FAO (2011) estimated the amount of edible FW combining data on food commodities reported in FAO food balance sheets (FBS) (FAO, 2017) and FW percentages collected by Gustavsson et al. (2013) from various sources, e.g. scientific literature and national authorities. Bräutigam et al. (2014) adopted FAO’s approach, considering the same waste coefficients, except for the “postharvest handling and storage”, calculated for each of the EU27 countries based on data reported in FBS. Vanham et al., 2015, Porter et al., 2016 considered as well FBS as source of data on food supply, but selected different FW percentages from literature. Particularly, Vanham et al. (2015) calculated the average FW generation in Europe, starting from the FW produced in six countries for which reliable data were provided, whereas Porter et al. (2016) made an average of more than one coefficient, when data were available, to overcome the possible limited representativeness and accuracy of some punctual coefficient reported by FAO (2011). The main difference between the abovementioned studies is the type of FW considered, namely avoidable or unavoidable, as explained in the following section. Stenmarck et al. (2016) considered data compliant with the FUSIONS framework, collected from part of the European Member States and scaled-up to the European level. Quality criteria were established for the inclusion of results in the overall assessment of FW generation at EU level. This is an important element when different sources of data are considered and combined to ensure consistency within the assessment. Tisserant et al. (2017) built a multiregional waste input-output model on the basis of the multi-regional environmentally extended supply and use table database Exiobase (Merciai et al., 2014). Alexander et al. (2017) estimated loss, waste and inefficiencies of the global food system considering literature data on the global cropland and grassland net primary production (NPP) and on inefficiencies, losses and waste coefficients. Particularly, for the consumption stage they referred to the coefficients reported by FAO (2011). They reported the results in four unit of measures, namely: wet mass, dry mass, energy content, and protein content. Kemna et al. (2017) depicted the overall flows characterising the food system, using various sources of data, such as FAO, Eurostat, the European Food Safety Authority (EFSA), and scientific literature.

### 3.4 Breakdown in product groups

Five out of ten studies reported a breakdown per food product or product group (Bräutigam et al., 2014, FAO, 2011, Porter et al., 2016, Vanham et al., 2015, Kemna et al., 2017), but only three of them reported explicitly waste coefficients, namely the percentages of inputs to a certain stage of the supply chain which end up to be FW (Table 3). Similarities in coefficients were observed between FAO, 2011, Porter et al., 2016 because the latter rely partly on FAO study (FAO, 2011). Main discrepancies, consisting in coefficients which were almost double, were observed for: cereals at agricultural production, marine products and milk at storage and handling stage, and for fruit and vegetables at distribution. The waste coefficients estimated by Vanham et al. (2015) for the consumption differed from the other two studies mainly for cereals, and roots and tuber food categories. This variability in coefficients does not affect the comparability of the study, but reflects variability and uncertainty in the estimation of FW generation, according, e.g., to the context and the season in which the estimation is carried out.

**Table 3:** FW percentage coefficients considered in the studies per food product group and per food supply chain stage. The breakdown proposed by Porter et al. (2016) was considered both for the supply chain and for the food product groups. (p) = processed product, (f) = fresh product, values in brackets represent the standard deviation of the mean, when available.

Food group	Agricultural production		Storage and handling		Manufacturing		Distribution		Consumption		
	FAO (2011)	Porter et al. (2016)	FAO (2011)	Porter et al. (2016)	FAO (2011)	Porter et al. (2016)	FAO (2011)	Porter et al. (2016)	FAO (2011)	Porter et al. (2016)	Vanham et al. (2015)
Cereals	2	4.33	4	3.85	0.5#, 10$	10.5	2	3.00 (1.00)	25	27	17.12 (8.6)
Fruit and vegetables	20	20	5	7.32 (5.32)	2	2	10 (f)	4.87 (2.49)(f)	19 (f)	19.00 (f)	26.2 (13.9)*
											25.5 (12)+
							2 (p)	2.00 (p)	15 (p)	15.00 (p)	
Marine	9.4	9.4	0.5	7.90 (7.40)	6	6	9 (f)	9.00 (f)	11 (f)	11.00 (f)	14.5 (6.5)
							5 (p)	5.00 (p)	10 (p)	10.00 (p)	
Meat	3.2		0.7		5	5	4	4.05 (0.05)	11	11	14.5 (6.6)
Bovine		2.3		0.63 (0.01)							
Mutton & Goat		10		0.59 (0.02)							
Pig		2.5		0.32 (0.09)							
Poultry		7		0.94 (0.82)							
Eggs	4	4	–	1.86 (1.94)	0.5^	0.5	2	2	8	8	11.9 (3.9)
Milk	3.5	3.5	0.5	1.67 (1.86)	1.2	1.2	0.5	0.82 (0.32)	7	7	7 (2.8)
Oilseeds & Pulses	10	5.28	1	1.15 (1.35)	5	5	1	1.00	4	4	5 (1.8)
Roots & Tubers	20	20	9	7.61 (4.61)	15	13.82 (1.18)	7	7.00 (f)	17	17.00 (f)	25.5 (14.2)
							3	3.00 (p)	12	12.00 (p)	

# Milling.

$ Processing.

^ Sum for the stages “storage and handling” and “processing”.

* Value for vegetables.

+ Value for fruit.

### 3.5 Reliability of estimates

Tostivint et al. (2016) identified five elements for the evaluation of the reliability of existing data on FW: quantification method, sampling procedures, scaling factor, stratification and weighting, and overall uncertainty around the estimates.

All the analysed studies were based on data collected at the national level from indirect measurements, e.g. surveys, statistics or a combination of the two. A systematic analysis of the abovementioned elements was not found in any of the documents, however, some of them reported considerations on the reliability of the estimates. Stenmarck et al. (2016) described the criteria adopted to scale-up national data to the European level per each stage of the food supply chain. Furthermore, they assessed the uncertainty associated with the scaling-up, estimating the 95% confidence interval. Monier et al. (2010) realised a plausibility check for the manufacturing phase – consisting in a comparison of data collected by Eurostat with the results of other studies – and found their results reasonably similar.

Besides, the examined studies reported some critical qualitative considerations on the weaknesses that may affect their results. Monier et al. (2010) highlighted that Eurostat data were collected by European Member States adopting different approaches, which may limit their comparability. Gustavsson et al. (2013) acknowledged that the FBS may be not completely reliable due to data gaps and highlighted that estimations for the agricultural stage, where a large part of the FW is ploughed into the soil, are characterised by a considerable uncertainty. Vanham et al. (2015) highlighted the importance of assessing the uncertainty associated to FW and assumed a normal distribution for waste coefficients, whereas Porter et al. (2016) reported the standard deviation of waste coefficients, when available. Alexander et al. (2017) made qualitative considerations on the uncertainty of the sources of data underpinning their estimation highlighting that the level of uncertainty of FAO coefficients (Gustavsson et al., 2013), used for their accounting, was difficult to define. Tisserant et al. (2017) reported that, being based on statistics on recorded waste flows, Exiobase likely underestimated actual waste flows due to “unregistered waste”.

Although quantitative estimation of uncertainty may be challenging due to the complexity and variability of food supply chains, considerations on reliability of data may support a better interpretation of results, the possibility of improving existing estimations, and their use in the decision-making process.

### 3.6 FW quantification at the global scale

**Fig. 2:**
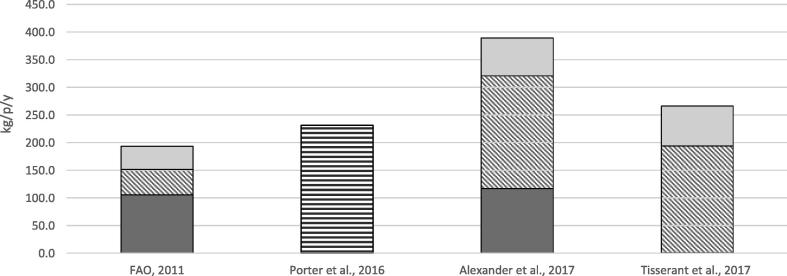
Global FW generation per capita and per year. FAO (2011) includes only edible fraction of FW.

Fig. 2 reports the estimated per capita and per year FW generation at the global level in terms of wet mass. The correspondence between the classification reported in each examined study and the classification of the life cycle followed in this study is described in Table 4. The manufacturing and distribution stages were reported in aggregated form because a breakdown for these stages was not available in Alexander et al. (2017). The contribution of agricultural residues and unharvested crops, animal metabolism, and over-eating estimated by Alexander et al. (2017) were not reported in Fig. 2 because they were not part of FW definition adopted in the present paper. Coherently with the Exiobase reporting conventions, FW in Tisserant et al. (2017) was expressed as dry mass. To make them coherent with the others, reported as wet mass, the amounts of FW were converted in wet mass, considering the default percentage dry matter content as calculated from the study by Alexander et al. (2017). However, it has to be highlighted that this assumption may influence importantly the results. Indeed, moisture content and wet mass are correlated by a function of the form “wet mass = dry mass/(1 − moisture content)”. Therefore, a limited variation of the moisture content within the range of reasonable values for FW can correspond to considerable change in the estimated wet mass. FAO results (2011) are not completely comparable with the others because they are accounting for the edible fraction of wasted food only. This may justify the lower results, particularly for the stages manufacturing and distribution, and consumption. Assuming negligible the influence of the reference year, the difference between the results by Porter et al., 2016, FAO, 2011 – in principle – should represent the inedible fraction of food, which correspond to about 16% of the total FW. However, the two studies are characterised by the adoption of different FW coefficients, which could have influenced the estimation. Estimated FW from primary production were considerably lower in the study by Tisserant et al. (2017) than in the ones by FAO, 2011, Alexander et al., 2017, who reached similar results. A possible explanation is that FW during primary production from plant-based products can be left in the field and, therefore, not be accounted for by waste statistics which are at the basis of the approach adopted by Tisserant et al. (2017). Beyond FW reported in Fig. 2, Alexander reported about 1680 kg/p/y of inefficiencies due to animal metabolism, and 61 kg/p/y of inefficiencies due to over-consumption.

**Table 4:** Correspondence between the stages of the supply chain considered in the present study and in the reviewed studies. The table is based on the terminology reported in original papers. n.c. = not considered.

	Primary production and post-harvest	Manufacturing	Distribution	Consumption
Monier et al. (2010)	n.c.	Manufacturing	Retail/Wholesale	– Households – Food Service/Catering
FAO, 2011, Gustavsson et al., 2013	– Agricultural production – Postharvest handling and storage	Processing and packaging	Distribution	Consumption
Bräutigam et al. (2014)	– Agricultural production – Postharvest handling and storage	Processing and packaging	Distribution	Consumption
Vanham et al. (2015)	n.c.	n.c.	n.c.	Consumption
Porter et al. (2016)	– Agricultural production – Storage and handling	Processing	Distribution	Consumer
Stenmarck et al. (2016)	Primary production	Processing	Wholesale and logistics and Retail and Markets	– Food service – Household
Alexander et al. (2017)	Losses from: – crops harvested	Losses from: – process commodities – animal products	n.c.	Losses from: – food consumption
Tisserant et al. (2017)	Agriculture	Food processing	Services	Households and government
Kemna et al. (2017)	Crops pre-processing, partitioning and export	Processing, wholesale and product import	Retail and products exports	Private households and food service

### 3.7 FW quantification at the European scale

**Fig. 3:**
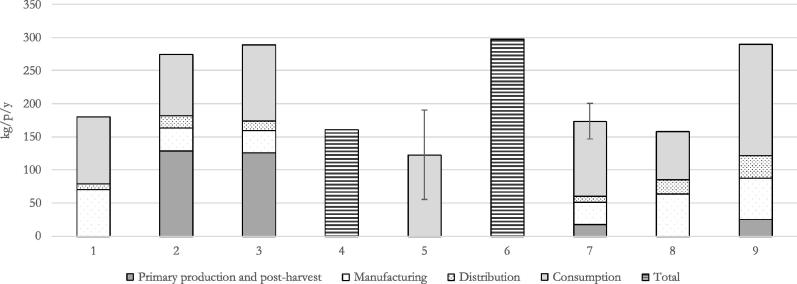
European FW generation per capita and per year and uncertainty (minimum and maximum value for 5: 95% confidence interval for 7), when reported in the study. 1: Monier et al. (2010); 2: FAO (2011); 3: Bräutigam et al. (2014); 4: data collected by Eurostat (2017b); 5: Vanham et al. (2015); 6: Porter et al. (2016); 7: Stenmarck et al. (2016); 8: Tisserant et al. (2017); 9: Kemna et al. (2017). FAO (2011) includes only edible fraction of FW.

Fig. 3 reports the amount of FW and waste generated at the European scale estimated by the examined studies.

As for FW generated globally, the studies based on FAO methodology, namely Bräutigam et al., 2014, FAO, 2011, reported a considerable amount of FW generation at primary production and postharvest, which was, instead, completely neglected by Monier et al. (2010) or only partly accounted for in the data collected by Stenmarck et al. (2016). As for global estimations, this difference may be justified by the fact that waste statistics do not account for the amount of FW which is not sent to waste management facilities. Kemna et al. (2017) found that discarded food from the agricultural phase used for animal feeding was about the same amount of FW generated at the same stage. Anyway, discarded food used as animal feed was not considered FW according to Kemna et al. (2017) and, is therefore, not reported in Fig. 3.

Kemna et al. (2017) specified the amount of water lost or added to food products along their life cycle, e.g. due to evaporation, whereas this element was not specified by the other studies and it is not clear whether the amount of lost or added water was accounted within FW volumes.

FAO, 2011, Bräutigam et al., 2014, and Stenmarck et al. (2016) reported a similar amount of FW for the manufacturing stage, although they include different types of materials. Indeed, FAO and Bräutigam et al. (2014) accounted only for edible fractions of food, whereas Stenmarck et al. (2016) included also inedible ones. Monier et al., 2010, Tisserant et al., 2017, and Kemna et al. (2017), who included in the accounting the inedible fractions of food, estimated a similar FW generation at manufacturing stage.

The distribution stage was found to generate a lower amount of FW than the other stages in all the analysed studies, varying from 9 kg/p/y to 38 kg/p/y.

**Fig. 4:**
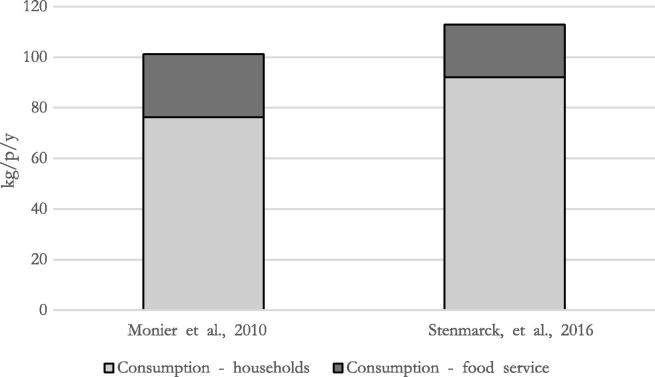
Food Waste occurring in households and food services.

The consumption stage was in the majority of the studies the main contribution, and the amount of avoidable FW ranged between 62% (Kemna et al., 2017) and 80% (Vanham et al., 2015). Furthermore, Monier et al. (2010), and Stenmarck et al. (2016) made a subdivision between FW happening in households and in food services (Fig. 4). Households resulted to be the main contributors to FW from consumption, ranging between 76 kg/p/y and 92 kg/p/y, whereas FW in food service was comprised between 21 kg/p/y and 25 kg/p/y. For the other studies, it was not possible to extrapolate such kind of breakdown, due, for example, to the fact that the FBS do not differentiate between food consumed in households and outside.

Stenmarck et al. (2016) reported the uncertainty associated with FW estimations per each stage, through the assessment of a confidence interval. The highest uncertainty was observed for the manufacturing stage, where the confidence interval was ±75% of the average value, whereas the confidence interval for the overall estimation was ±16%.

## 4 Discussion and open issues

The reviewed studies reported that FW generation along the supply chain ranged between 194 kg/p/y and 389 kg/p/y at the global level, and between 158 kg/p/y and 298 kg/p/y when referring to the European scale. The highest share of FW was in most cases produced at consumption stage, followed by the food manufacturing one. However, it was also observed that estimations for the manufacturing stage were quite uncertain and further in-depth analysis would be advisable.

Differences between the studies were due to the combined effects of different variables, which covered quantification approaches and sources of data, and to related uncertainties. These differences may in same cases have led to not directly comparable results. Anyway, the purpose of the present study was not a mere comparison of the results, but an assessment of how differences in accounting approaches and sources of data may influence the estimation.

Coherently with FW definitions and data sources considered, quantification approaches captured different FW streams. FAO (2011), for example, accounted for the edible fraction of food not eventually eaten by humans, whereas Monier et al. (2010) considered both edible and inedible parts of food but focused only on the collected FW and on flows treated in waste management facilities. The high variability observed for the results on primary production, for example, may originate from differences in quantification approaches, considering that FW at this stage may not be sent to waste treatment facilities and, therefore, not be captured by waste statistics. Furthermore, accounting only for the edible fraction of food, as in FAO study (2011), generated more relevant differences above all for the stages of the supply chain in which a higher amount of inedible FW is produced, such as manufacturing (Beretta et al., 2013). Further considerations on the importance of distinguishing between edible and inedible fractions of FW are reported in the following section, with a focus on support of FW estimations to European policies.

Almost all the studies analysed were based on different sources of data, which may imply the combination of different statistical and literature sources originally meant for other purposes (e.g. Kemna et al., 2017). Due to the complexity of the food chain and the multitude of possible estimations approaches, the consistency between different sources should always be considered, e.g. by mean of quality criteria, as done by Stenmarck et al. (2016).

The choice of waste coefficients covered a central role in quantification approaches taking food supply as starting point for FW estimation, such as the ones adopted in FAO (2011), and Vanham et al. (2015). Canali et al. (2016) highlighted that several drivers, including intrinsic characteristics of food products and socio-cultural aspects, influence FW generation. Capturing such variability through waste coefficients is a challenging task, above all when considering a broad geographic scale, such as global or European. Indeed, coefficients are often taken from studies based on direct measurements, which may not be representative of an entire country or world region (Xue et al., 2017). Therefore, the more detailed is the breakdown of the analysed geographical area and of the food commodity groups, the highest is the representativeness of waste coefficients. A possible option to increase the robustness of the waste coefficients, applied by Porter et al. (2016), is to average different values referred to the same geographical area and food commodity group, taking into account their variability, but this may not always possible due to lack of data. Efforts to broaden the coverage of FW based on direct measurement are, therefore, needed, not only for small-scale estimations, but also to strengthen the quantification of FW generation at European and global scales.

Furthermore, the analysed estimations, as all types of FW quantification exercises, were characterised by uncertainties. Uncertainties may be due to various sources, which, for example, comprehend biases in measurement and limited representativeness of FW coefficients (Hanson et al., 2016). All the analysed studies made considerations on the reliability and uncertainty of their results, however a quantitative estimation of the uncertainty of FW quantification was reported only in two studies (Stenmarck et al., 2016, Vanham et al., 2015). Taking track of the uncertainty of FW estimations is advisable since it increases the awareness on the results of users, fostering their use in decision-making processes and allowing them to improve the quality of data. The “food loss and waste accounting and reporting standard” (Hanson et al., 2016) reports an extended list of the potential causes of uncertainties for FW quantification and provides guidance on how to consider uncertainty qualitatively and quantitatively. An alternative way for assessing the quality of data sources was followed in the study on FW quantification and potential for reduction in Switzerland by Beretta et al. (2013), who used the pedigree matrix, originally developed by Frischknecht et al. (2007) for inventory data of the ecoinvent database.

### 4.1 FW estimations for supporting European policies and interventions

FW prevention, reduction and valorisation are targets addressed by European policies. The waste framework directive (European Parliament and European Council (2008) amended by European Parliament and European Council (2018)) proposed a hierarchy for waste prevention and management, which considers prevention as the priority option, followed by re-use, recycling, other types of recovery and disposal. Moreover, with the European Circular Economy Action plan (EC, 2015), the European Commission committed to define a common methodology for FW measurements, as well as to achieve the target on FW reduction foreseen by the SDG 12.3.

**Fig. 5:**
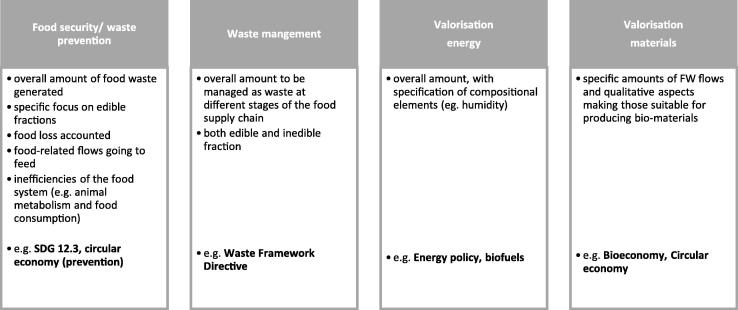
Overview of different perspectives adopted for the accounting of FW and inefficiencies of the food system, and related policies.

FW accounting may serve different policies. Specific aspects are discussed within the following sections in relation to: (i) FW waste prevention, FW management and the link to food security; (ii) valorisation of FW as material and energy, and the link with circular economy. An overview is provided in Fig. 5.

#### 4.1.1 Food waste prevention, food waste management, and food security

Different drivers cause FW generation along the supply chain (Roodhuyzen et al., 2017). Therefore, the breakdown in life cycle stages is useful to identify both specific hotspots and targeted reduction strategies (Priefer et al., 2016). Furthermore, the intrinsic characteristics of FW streams may influence the applicability of the actions foreseen by the waste hierarchy. In particular, the distinction between avoidable and unavoidable FW is an important information to assess the potential for prevention and the implications to food security. The focus of FW prevention is on the avoidable part because it can – per definition- be prevented contributing to reach the objective of halving FW by 2030 (EC, 2015). The distinction between avoidable and unavoidable FW can be influenced by cultural and behavioural aspects and it is not straightforward. Edibility may not always be associated with avoidable FW. Some fruit or vegetable peels, e.g. apple or potato peels, may be edible, in the sense that they are suitable for human consumption and have a nutritional value, and, at the same time, be considered unavoidable FW in contexts where they are not generally eaten. Hence, the distinction between avoidable and unavoidable should be coupled with a clear definition of which types of FW fall under the two categories to support FW quantification, as recently performed for fruit and vegetables by De Laurentiis et al. (2018). Avoidable FW is most likely happening at the consumption stage. However, manufacturing may be as well a source of avoidable FW due to non-optimal organization and coordination between actors, and to consumers’ expectations on a wide availability of products (Beretta et al., 2013).

Another element that deserves to be discussed is the unit of measure in which FW amount are reported. FW estimations were expressed in most of the analysed studies as actual mass, which is the most direct way of measuring FW, e.g. through direct weighting. Conversion in dry mass may be calculated through moisture content coefficients. However, it has to be noted that FW are characterised by a moisture content ranging on average between 70% and 80% (Kumar et al., 2010) and even small changes in the moisture content coefficient may influence importantly the overall mass of FW. Expressing FW either in dry or wet mass may be advantageous depending on the aim of the estimation. Moisture content of food can change considerably along its life cycle, e.g. during manufacturing and cooking. Therefore, reporting FW as dry mass is more appropriate to identify inefficiencies along the supply chain. Alexander et al. (2017), for example, found that processing of primary crops resulted in an important net loss of water, which may be accounted as FW in other studies, but cannot be considered as a real inefficiency of the food system or a source of FW. On the contrary, wet mass is indicative of the amount of FW that has actually to be managed, transported and treated and it is, therefore, more meaningful when planning strategies for FW management and assessing the environmental or economic burden. Couple information on FW amounts with moisture contents and amount of water added/lost along the food supply chain is an advisable approach to give an effective support to policy making.

Besides, Alexander et al. (2017) included in their estimation two elements, animal metabolism and over-consumption, which do not comply with the definition of FW as intended in this paper and defined in the Introduction. However, the study highlighted interesting elements for the purpose of policies addressing food security. Indeed, animal metabolism represented by far the largest inefficiency of the food system, after crop residues left on the field, and was one order of magnitude bigger than the streams of FW considered in the present study, confirming that decreasing the consumption of animal-based product could importantly contribute to the reduction of the resource intensity of the entire food system. Furthermore, the extent of over-consumption resulted to be almost equal to FW during the consumption stage. Initiatives aimed at contrasting food consumption above nutritional needs, should be part of the discussion on food availability and security.

#### 4.1.2 Food waste valorisation as energy and material, and circular economy

Acknowledging that the prevention of avoidable food waste generation should be the main target to be achieved, it is as well important to understand to which extent unavoidable fractions of FW may help the transition towards a circular bio-based economy (EC, 2017b). Options for the valorisation of unavoidable FW at the global and European scales include, for example, the extraction of high-valuable compounds, the use as animal feed, the production of bio-materials and the generation of biofuels. Valorisation is, generally, more applicable when there is homogeneity of the waste flows (Girotto et al., 2015).

The separation of edible and inedible fractions of food may happen at different points of the supply chain, such manufacturing or consumption, depending on the form in which the food is consumed (Bernstad et al., 2017). Homogeneous FW streams are most likely generated at the manufacturing stage, where FW can be collected separately (De Laurentiis et al., 2018). In households and food services, instead, FW is generally managed as organic waste or mixed municipal waste, except for particular FW categories, such as used cooking oils. However, beyond enhancing the potential for FW valorisation, processed food may require more packaging or energy for storage than fresh food, increasing environmental burden. Therefore, a broad perspective, including the analysis of possible environmental offsets, should be adopted when analysing scenarios for FW valorisation.

Two out of ten of the analysed studies reported the distinction between avoidable and unavoidable FW at the consumption stage (Kemna et al., 2017, Vanham et al., 2015). Other studies, performed at smaller geographical scales, such as Quested et al., 2013, Beretta et al., 2013, provided the amount of avoidable and unavoidable FW produced along the stages of the supply chain. These values may be used as proxies for the European context. Anyway, further investigations are advisable.

Another element influencing the relevance of FW accounting to support circular economy strategies is the type of material considered as FW. Within the FAO study (2011), for example, only the edible fraction on food is accounted, therefore the results did not capture the potential for valorisation of inedible parts of food. Furthermore, from a circular economy perspective, not all the valorisation pathways have the same value and options increasing the cascading use of resources are, in principle, preferable (Corrado and Sala, 2018). Food discarded along the supply chain and recycled for other purposes, e.g. animal feed, does not often comply with FW definition and falls under the category “by-product”. However, it would be interesting to know which the amount of recycled by-products is. On one hand, there is the need to know the potential of valorisation not yet disclosed and, on the other hand, to assess if preferable valorisation options are practicable. Kemna et al. (2017) were the only ones estimating the amount of by-products used as animal feed. It was equal to about 100 megatons and it was a considerable amount of material compared to the overall FW generation equal to 150 megatons. Notwithstanding these estimations are considered to be highly uncertain, they are highlighting important biomass flows to be accounted for in the overall food system. Other valorisation options, such as the use in bio-refineries, were not considered in the study of Kemna et al. (2017) study possibly because they are still little practiced in Europe.

## 5 Conclusions

The present study highlighted that available data provide an overall picture on FW generation at global and European scales, but are not enough to support the definition of specific FW-related interventions and the monitoring of their progress over time. Indeed, despite being all the studies based on indirect measurements, they are built on different quantification approaches and data sources, and are characterised by uncertainties. All these elements made, in some cases, the average results diverging. Differences between the results are not always fully interpretable. Therefore, a robust estimate of FW at European and global level is currently not available. Moreover, existing estimations partly lack important information for the accomplishment of specific FW interventions and policies.

Only two out of ten studies analysed provided information on the avoidability of FW but focused only on the consumption stage in Europe. This kind of information is important to define strategies for FW reduction, according to the European hierarchy for waste prevention and management, which sets waste prevention as preferable option. In case of FW, prevention can be applied to avoidable fractions, whereas other management patterns should be followed for the unavoidable ones. The breakdown of the supply chain in stages, and of food in products or commodity groups are additional pieces of information, provided only by some of the analysed studies, which are important for the definition of FW reduction strategies. Indeed, on the one hand, they allow making more precise estimations and, on the other, they support a better identification of hotspots, potential targets of FW strategies. Furthermore, increasing the availability of quantitative information on uncertainty of results, reported only in two out of the ten studies, would be beneficial for their use in decision-making processes and for further refinement of estimations. Studies based on direct measurements may contribute to strengthen FW estimations at broad scale, providing punctual pieces of information, which have to be combined to define the overall picture.

The high variability in results and in methodological approaches detected in this study highlighted the need of additional and joint efforts to improve availability, reliability and level of detail in data on FW generation. Specifically, private and public decision makers should clearly define the policy objectives (e.g. prevention, valorisation, waste management, food security) to optimise efficiency and efficacy of data collection. The designing of a specific framework for data collection and their elaboration may benefit from the expertise on FW of research institutions. Finally, the actors of the food supply chain, e.g. farmers, industries and consumers, have a key role in providing data on FW generation.
